# Modular hip exoskeleton improves walking function and reduces sedentary time in community-dwelling older adults

**DOI:** 10.1186/s12984-022-01121-4

**Published:** 2022-12-30

**Authors:** Chandrasekaran Jayaraman, Kyle R. Embry, Chaithanya K. Mummidisetty, Yaejin Moon, Matt Giffhorn, Sara Prokup, Bokman Lim, Jusuk Lee, Younbaek Lee, Minhyung Lee, Arun Jayaraman

**Affiliations:** 1 Max Näder Lab for Rehabilitation Technologies and Outcomes Research, Shirley Ryan AbilityLab, Chicago, IL USA; 2 Departments of Physical Medicine and Rehabilitation, Medical Social Sciences and Physical Therapy and Human Movement Sciences, Northwestern University, Chicago, IL USA; 3 WI Robotics Co, Cheonan, South Korea; 4 Department of Radiology, University of California, San Francisco, USA; 5 Samsung Electronics Co, Suwon, South Korea

**Keywords:** Exoskeleton, Gait training, Clinical outcomes, Fall prevention, Aging

## Abstract

**Background:**

Despite the benefits of physical activity for healthy physical and cognitive aging, 35% of adults over the age of 75 in the United States are inactive. Robotic exoskeleton-based exercise studies have shown benefits in improving walking function, but most are conducted in clinical settings with a neurologically impaired population. Emerging technology is starting to enable easy-to-use, lightweight, wearable robots, but their impact in the otherwise healthy older adult population remains mostly unknown. For the first time, this study investigates the feasibility and efficacy of using a lightweight, modular hip exoskeleton for in-community gait training in the older adult population to improve walking function.

**Methods:**

Twelve adults over the age of 65 were enrolled in a gait training intervention involving twelve 30-min sessions using the Gait Enhancing and Motivating System for Hip in their own senior living community.

**Results:**

Performance-based outcome measures suggest clinically significant improvements in balance, gait speed, and endurance following the exoskeleton training, and the device was safe and well tolerated. Gait speed below 1.0 m/s is an indicator of fall risk, and two out of the four participants below this threshold increased their self-selected gait speed over 1.0 m/s after intervention. Time spent in sedentary behavior also decreased significantly.

**Conclusions:**

This intervention resulted in greater improvements in speed and endurance than traditional exercise programs, in significantly less time. Together, our results demonstrated that exoskeleton-based gait training is an effective intervention and novel approach to encouraging older adults to exercise and reduce sedentary time, while improving walking function. Future work will focus on whether the device can be used independently long-term by older adults as an everyday exercise and community-use personal mobility device.

*Trial registration *This study was retrospectively registered with ClinicalTrials.gov (ID: NCT05197127).

**Supplementary Information:**

The online version contains supplementary material available at 10.1186/s12984-022-01121-4.

## Background

The number of adults in the United States over the age of 65 will reach 80.8 million by the year 2040 [1], and the world population could reach two billion by 2050 [2]. According to the World Health Organization, the global average life expectancy at birth increased by 6.5 years (from 66.8 to 73.3) from 2000 to 2019, while the healthy life expectancy only increased by 5.4 years (from 58.3 to 63.7) [3]. This means that worldwide, we can expect a growing population of adults over 65 who are facing serious health challenges. This aging population faces an increased risk of disability, dependency, and increasing mortality mainly due to the natural aging process which involves age-related decline in multiple physiological process at the cellular and tissue level, called frailty [2].

Current research strongly suggests that exercise programs implemented in the older adult population are beneficial in decelerating age-related changes to physical and cognitive health, regardless of the age when exercise programs are initiated [4, 5]. Various exercise interventions have proven to be beneficial [6]. The nature of these traditional exercise interventions varies in term of components (resistance, stretching, strength, flexibility, balance, activities of daily living), setting (facility, home), delivery (individual, group, unsupervised, semi-supervised, supervised), duration, and frequency. Many types of physical activity will produce health benefits for older adults, but the prevalence of inactivity increases with advancing age: 25% of adults aged 50 to 64 years old are inactive, 27% of adults 65–74 years old, and 35% of adults 75 years and older [7].

Modern wearable robotic exoskeletons are powerful emerging technologies that are versatile enough for personalized patient-centered care, can be specifically designed to deliver multicomponent exercise interventions, and have been extremely beneficial in neurologically impaired clinical populations [8, 9]. However, the efficacy of robotic exoskeleton-based exercise intervention in a community setting is yet to be assessed in any population, especially the older adult population. Successfully deploying a rehabilitation exoskeleton exercise program outside of a rehabilitation clinic requires lightweight, easy-to-use exoskeletons, and realistic outcome measures related to real-world performance and functional improvements. Therefore, the overarching goal of this preclinical investigation was to evaluate the safety and efficacy of a novel exercise training protocol using a robotic exoskeleton in a cohort of otherwise healthy older adults in their own community setting.

In order to achieve this goal, two main knowledge gaps needed to be addressed. First, most existing robotic exoskeletons are designed to provide powered assistance that caters to a specific disease state, impairment, or disability. Healthy aging can result in a wide range of gait irregularities that generally lead to unsteady gait that is sensitive to external perturbations [10, 11]. Since these individuals are more vulnerable to external perturbations, a slight mismatch between their intended motion and the devices’ provided assistance can lead to the users adopting a more cautious gait or having a fall-like event. Both of these outcomes are likely to reduce the user’s confidence in the exoskeleton, which could negate the purpose of using an exoskeleton device for exercise or mobility intervention. To address this issue, we utilized a Delayed Output Feedback Controller (DOFC) which directly responds to the wearer’s hip kinematics and has been shown to stabilize oscillatory systems under certain conditions while avoiding unexpected torque changes that could perturb older adults’ gait [12, 13]. Our preliminary work with this technique (using single training sessions) demonstrated the gait and metabolic benefits of using a lightweight modular hip exoskeleton in healthy young individuals [14–17], and agreed with contemporary research that has shown that hip exoskeletons provide a greater metabolic cost savings than ankle or knee exoskeletons [18]. We believe this feedback control strategy will prove beneficial to a wide range of age-related gait irregularities. Second, most robotic systems are built for use in a controlled laboratory or clinical setting and hence are relatively cumbersome, both in terms of physical mass and operational complexity. An exoskeleton like the Gait Enhancing and Motivating System for Hip (GEMS-H; Samsung Electronics Co., Suwon, South Korea) that is easy-to-transport, easy to don and doff, and user friendly was needed for a community-based intervention. However, there was very limited information available on implementing and validating efficacy of robotic exoskeleton-based exercise interventions in a real-world environment outside the clinic or laboratory setting [18]. Thus, this preclinical investigation addressed these overarching goals by administering in-community gait training with an exoskeleton for the first time. These findings will serve as a useful guide for future large scale translational clinical trials investigating the impact of community-based exercise interventions in older adults using robotic exoskeletons.

Twelve otherwise healthy adults over the age of 65 underwent a novel walking training intervention involving twelve 30-min sessions using a modular hip exoskeleton in the common areas of their senior living community. Participants underwent supervised training for a series of activities that were achievable within their own community (including level ground, inclines, stairs, and activities of daily living) and did not require any additional equipment besides the exoskeleton. Their training progression followed a model of increasing resistance and decreasing assistance over the course of the twelve sessions. The external assistance and resistance were personalized to each participant’s ability in order to challenge them at an appropriate level. Outcome measures related to gait performance, patient-reported questionnaires, and sedentary bouts/day were collected at pre- and post-intervention time points. *Hypothesis:* Novel robotic exoskeleton-based training intervention will lead to significant walking functional benefits and decreased sedentary time.

## Methods

### Study design

This study was designed to assess the safety and efficacy of using a bilateral hip-based exoskeleton to improve gait function in community dwelling older adults. We utilized both the assistive and resistive modes of the device to personalize the intensity of exercise and to provide an active recovery period. Twelve participants over 65 years old completed a total of twelve 30-min gait training sessions over a period of 4–6 weeks using the GEMS-H. All gait training sessions were completed in the community spaces at The Merion, a senior living community located in Evanston, Illinois.

### Functional outcome measures

Outcomes were collected at two separate time points in order to compare pre- and post-intervention status. Functional outcome measures included five times sit-to-stand (5xSTS), ten meter walk test (10MWT), six minute walk test (6MWT), Berg-Balance Scale (BBS), and functional gait assessment (FGA). Patient-Reported Outcomes included the Quebec User Evaluation of Satisfaction with assistive Technology (QUEST). These outcomes were selected based on their common use in prior literature assessing gait function, balance, endurance, and device satisfaction.

### Activity monitoring

Each participant’s activity levels were monitored using an ankle-worn sensor (ActiGraph wGT3X-BT; ActiGraph LLC., Pensacola, FL). For sedentary analysis, participant’s activity was monitored for six days prior to the baseline session, and for 6 days following the final assessment. ActiGraph recorded 3D-acceleration data at a sampling frequency of 30 Hz. Raw acceleration data is analyzed using ActiGraph’s proprietary software (ActiLife 6.13).

### Sedentary time/inactivity analysis

Each participant was issued an ActiGraph system and instructed to wear the device at all times for six consecutive days before and after the training intervention. This data was analyzed using a 10-s actigraph epoch data format and data were subjected to the Choi algorithm for wear time and non-wear time separation [19]. Further, ActiGraph data was only analyzed for sedentary behavior between 5 AM and 11 PM, assuming most physical activity happens during waking hours, and this was verified with individual reports. Active to sedentary transition was characterized by a boundary condition of three or more minutes of inactivity. This analysis resulted in both the number and duration of sedentary bouts each day, where a bout duration is defined as the number of consecutive minutes spent in a sedentary state. After six consecutive days of data collection, the average daily bout number and duration were calculated. These daily averages from the PRE intervention state to the POST intervention state were compared using a one-tailed paired *t-*test and $\alpha$=0.05. The sedentary analysis procedures were based on literature related to aging research [20, 21].

### Participants and inclusion/exclusion criteria

Twelve individuals above the age of 65 years old were recruited to participate in twelve sessions that occurred 2–3 times per week over a 4–6 week period using the GEMS-H. In addition to selecting individuals over the age of 65, qualifying participants also had to be able to walk with or without an assistive device for greater than three meters. Medical clearance was obtained from each participant’s primary physician prior to training with the device. Participants were excluded from recruitment if they were unable to comprehend or provide consent, were unable to physically fit within the device, or had any significant neurological diagnoses that would impact safe use of the device.

### Hip assist exoskeleton and personalized tuning

**Fig. 1:**
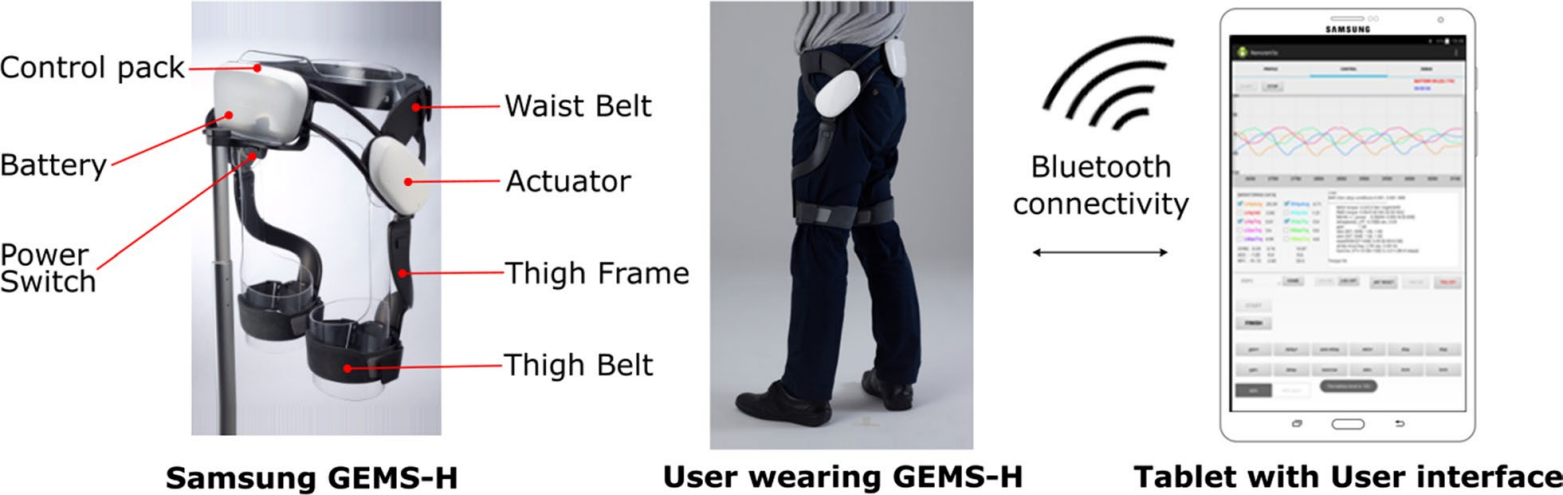
The GEMS-H exoskeleton has several modules which can be used to customize the device to different body types. The base unit (control pack, battery, waist belt, and actuator) comes in three sizes, and can be further adjusted to hip width. The thigh frames and thigh belts are also modular and adjustable, ensuring effectiveness and comfort for a variety of leg dimensions. The user interface allows control parameters to be customized to the user’s preference, and to accommodate a range of aging related gait irregularities

The GEMS-H is a hip-based robotic exoskeleton worn around the waist and fastened to the thighs to assist with hip flexion and extension (Fig. 1). The GEMS-H device has a pair of actuators that generate assistive or resistive forces at each hip joint. The device weighs 2.1 kg and comes in three sizes. The width of each version can be adjusted to fit individual body size. There are magnetic joint angle sensors in each hip of the exoskeleton to continuously track the user’s kinematics and provide feedback to the controller which stabilizes assistance/resistance based on instantaneous user needs.

### DOFC for gait assistance and resistance

**Fig. 2:**
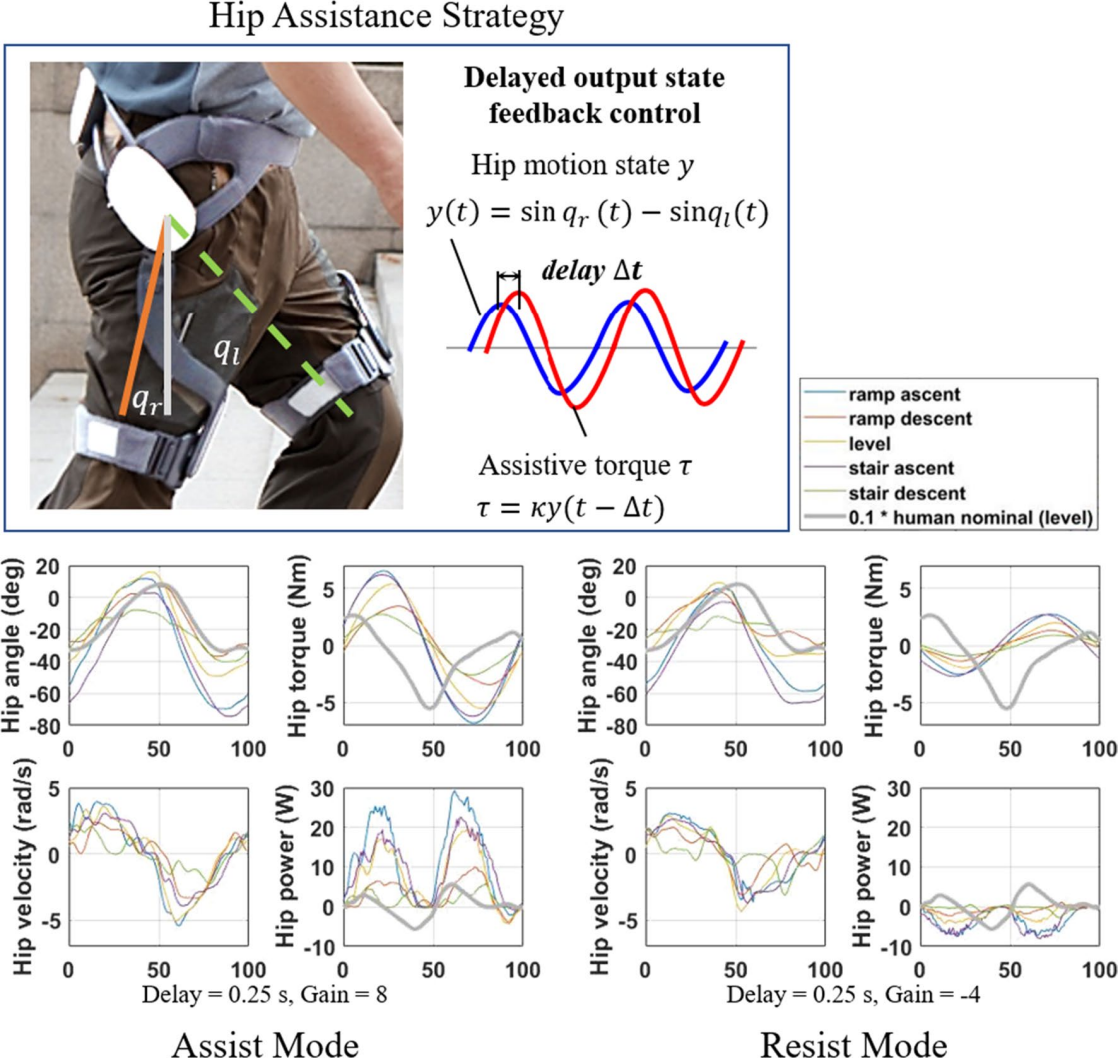
The GEMS-H uses the DOFC to prescribe torque inputs into both hip joints (top). Without the need for explicit activity recognition or gait phase estimation, it can provide appropriate torque and power for a variety of walking tasks during both assist mode (bottom left) or resist mode (bottom right). For comparison, the grey lines in each figure show human nominal values (scaled down by multiplying by 0.1, and assuming a body weight of 75 kg at level ground) based on data made available by Bovi et. al [23]. Note that the exoskeleton’s definition of gait cycle percentage may differ slightly from this reference source, and that the GEMS-H does not measure joint angles as accurately as the motion capture system used in [23]

The GEMS-H implements a simple self-excited DOFC method to generate assistive and resistive torques. As shown in Fig. 2, the DOFC method does not include a gait phase estimator or a reference lookup for generating torque profiles, yet it can be generalized to operate under various walking speeds and environments (e.g., stairs and ramps) without the need for task-specific parameter adjustments [12, 13]. The assistance/resistance torque is immediately applied following the movement of the user by reflecting the change of leg motion from reading the wearers hip kinematics at every control period (= 0.01 s i.e. 100 Hz) [12, 13]. This time delayed, self-feedback controller approach is known for stabilizing oscillatory systems like human locomotion, and can be generalized to operate under various walking speeds and environment (e.g., stairs and ramps) without the need for task-specific parameter adjustments [12, 13]. The magnitude of assistance/resistance can be varied over a range of values to personalize the external assistance/resistance to every user’s self-chosen comfort level. An in-depth description of this controller and earlier hardware design are described in previously published manuscripts [12, 13, 22].

As the current study is the first time older adults trained with this exoskeleton, we required the engagement of clinicians to train our participants to safely use the device. Thus, based on clinician feedback and practicality of using in real-world settings, we chose two control parameters that the clinicians could tune to personalize the device setting for each user. These are the feedback gain $\kappa$(positive gain for assistive torque and negative gain for resistive torque respectively) the feedback time delay $\Delta t$. The magnitude of gain determines the strength of the assistive/resistive torque generated. The combination of these two settings will determine the type of torque generated, i.e. assistive or resistive, and the timing of torque input with respect to the gait cycle.

### Smoothing hip motion state with low pass filter

Figure 2 show the hip assistance and resistance strategies with DOFC framework. We define an output state ${y}_{\mathrm{raw}}$ as the ground projected leg motion:

1 $${y}_{\mathrm{raw}}\left(t\right)=\mathrm{sin}{q}_{\mathrm{r}}\left(t\right)-\mathrm{sin}{q}_{\mathrm{l}}(t),$$

where ${q}_{\mathrm{r}}$ and ${q}_{\mathrm{l}}$ are the right and left joint angles, respectively. For both terms, hip extension is considered a positive angle.

The original noisy output state is smoothed by passing it through a simple first-order low-pass filter (also known as an exponential moving average filter):

2 $${y}^{\mathrm{cur}}={(1-\alpha )y}^{\mathrm{prv}}+\alpha {{y}_{\mathrm{raw}}}^{\mathrm{cur}},$$

where ${y}^{\mathrm{cur}}$ denotes the currently smoothed output state, ${y}^{\mathrm{prv}}$ is the previously smoothed state, ${{y}_{\mathrm{raw}}}^{\mathrm{cur}}$ is the currently sensed original state, $\alpha$ is the smoothing factor. The current smoothed state ${y}^{\mathrm{cur}}$ is expressed as a weighted sum of the previous sample time state ${y}^{\mathrm{prv}}$ and the original state value of the current sample time ${{y}_{\mathrm{raw}}}^{\mathrm{cur}}$, and the smoothing rate can be modified by changing the smoothing factor. The − 3 dB cutoff frequency (the frequency over which the signal power is halved, denoted ${f}_{c}$), is given by this equation for discrete time systems:

3 $${f}_{c}=\frac{{f}_{s}}{2\pi }{\mathrm{cos}}^{-1}\left(1- \frac{{\alpha }^{2}}{2(1-\alpha )}\right),$$

where ${f}_{s}$ is the sampling frequency. The smoothing factor $\alpha$ = 0.05 in (2) was selected to generate the smoothed interaction torque. Combined with our sampling rate ${f}_{s}=100 Hz$, the − 3 dB cutoff frequency is 0.8165 Hz.

### Assistive or resistive torque generation from delayed feedback state

The assistive or resistive torque $\tau$ is generated through a combination of appropriate time delays $\Delta t$ and positive (assistive) or negative (resistive) gains $\kappa$:

4 $$\tau \left(t\right)=\kappa y\left(t-\Delta t\right)=\left\{\begin{array}{c}\kappa >0, {\text{assist mode}}\\ \kappa <0, {\text{resist mode.}}\end{array}\right.$$

The base control strategy in Fig. 2 can be extended for both right/left hip torque generation ${\tau }_{\mathrm{r},\mathrm{des}}$, ${\tau }_{\mathrm{l},\mathrm{des}}$ by modifying the original torque equation in (4).

The terms for right hip flexion, left hip extension are:

5 $${\tau }_{\mathrm{r},\mathrm{des}}\left(t\right)=-\tau \left(t\right)$$

$${\tau }_{\mathrm{l},\mathrm{des}}\left(t\right)=\tau \left(t\right)\cdot \delta ,$$

whereas for left hip flexion, right hip extension:

6 $${\tau }_{\mathrm{l},\mathrm{des}}\left(t\right)=\tau \left(t\right)$$

$${\tau }_{\mathrm{r},\mathrm{des}}\left(t\right)=-\tau \left(t\right)\cdot \delta ,$$

where $\delta$ denotes the hip extension-flexion torque ratio. Torque in the direction of hip extension is considered positive. The extension-flexion ratio $\delta =$ 1 was used in this study to generate equal hip extension and flexion torque strength [13].

### Device interface

The device is controlled through a custom built application on a hand-held tablet. Through the application, the trained physical therapist assisting each participant is able to turn on/off torque, switch between assist and resistance modes, and modify the gain ($\kappa )$ and delay ($\Delta t$) parameters. Gain increases or decreases the amplitude of assistance or resistance. The maximum value for gain in assistance mode is 15 (about 12 Nm peak torque), while the maximum (absolute) value in resistance mode is − 5 (about − 4 Nm peak torque). Delay allows the assistance or resistance to be applied earlier or later in the gait cycle. The range of delay is between 0.15 and 0.25 s. The tablet also displays real time information such as joint angle and torque values.

### Training progression

The GEMS-H training program was based on prior systems designed to improve the walking performance of the user [24, 25]. For this study, training sessions were conducted in regions of the participant’s community living facility, including indoor hallways, ramps, curbs, stairs, and multi-terrain surfaces. Older adults traditionally struggle with motivation to walk due to physical impairment and fear of falling. To encourage walking in the community environment, the physical therapists alternated the exoskeleton settings between resistance torque to help strengthen muscles and assistance torque to provide an active rest between resistance training. Seated breaks were allowed as necessary, but assistance mode was preferred when possible. Every subject’s first training session had at least ten minutes of resisted walking during their 30-min training sessions (in accordance to their ability), but the physical therapists sought to increase the time in resistance mode gradually throughout the 12 sessions.

Physical therapists were also able to adjust the resistance gain, assistance gain, and time delay to help tune the device to an appropriate resistance/assistance level and match the user’s gait. These parameters would change over the course of the twelve training sessions to match the subject’s change in gait, strength, or endurance. Priority was placed on increasing resistance and decreasing assistance during each new training session, in order to encourage progressive training.

After selecting the resistance time and the assist/resist gains, activities during each session were selected and modified based on the subject's ability to perform them successfully while providing a challenge. Activities were selected based on the following progression of increasing difficulty: level ground walking, speed changes, multi-directional/backward stepping, inclines/ramps, stair climbing, and obstacle negotiation. Obstacle negotiation included stepping over, weaving between, or stepping onto selected obstacles. Following this protocol, subjects 2 and 10 were additionally challenged by not always using their assistive devices (a cane and rolling walker, respectively) during the walking training, see Additional file 1: Table S1 for more details. All training was done under the supervision of a trained physical therapist, who would guard participants to prevent falls, particularly in the case that the subject’s regular assistive device was not being used.

### Statistical analysis

All outcomes/values are presented as mean ± standard deviation (SD), and the alpha value was set at *P* < 0.05 for indicating significance, and unless otherwise noted normality assumptions were checked and appear reasonable. Two-tailed paired *t* tests were used to compare the outcomes from pre and post testing. SigmaPlot 14.0 (Systat Software Inc., San Jose, CA, USA) was used to perform all statistical analyses.

## Results

### Training progression control parameters

All subjects spent at least ten minutes in resist mode during their first training session, but some spent more if they were deemed capable of doing so. The group averaged 14.2 min of resistance training in 30 min for the first session (the remainder was spent in assist mode). At the final (twelfth) training session, every subject was spending at least five more minutes in resist mode, with an average of 24.8 min out of 30. Please see Additional file 1: Table S2 for a full breakdown per subject.

During the first session, our physical therapists selected assistance gains, resistance gains, and time delays that were appropriate for each subject. The goal was to provide comfortable use, sufficient challenge during resistance, and sufficient help during assistance. A time delay of 0.25 s was selected for all subjects, and this value was held constant for all training sessions. Subjects started with an average assistance gain of 6.8, and resistance gain of − 3.4. Over the course of the study, the assistance gains were decreased and resistance gain magnitude increased for almost all subjects, for a final result of assistance gain of 3.4 and resistance gain of − 4.3 during the last training session (a more negative resistance gain produces more resistance torque). Please see Additional file 1: Table S2 for a full breakdown per subject.

### Functional walking performance

**Fig. 3:**
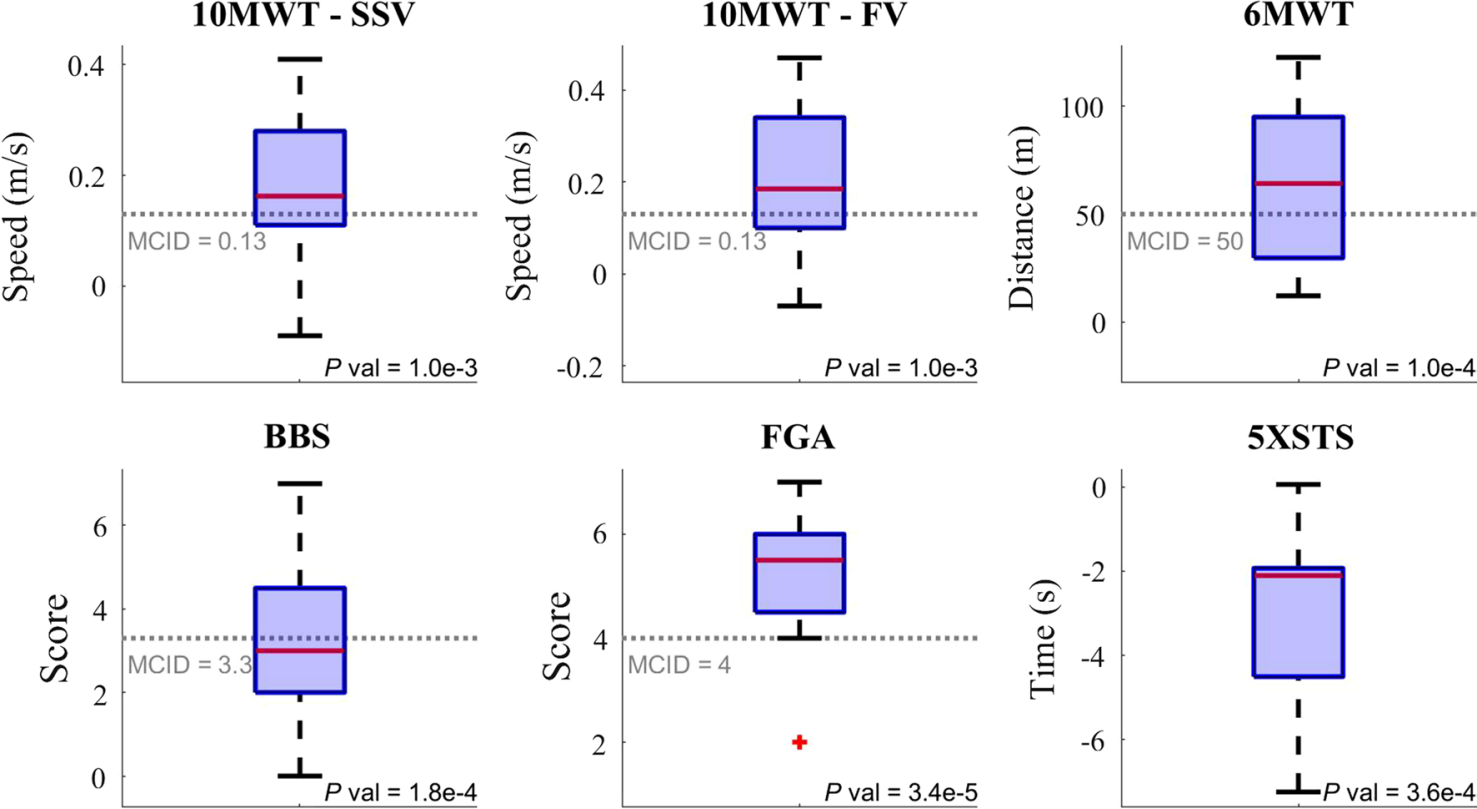
Change in performance-based outcome measures from pre- to post-intervention, compared to the MCID when available. Each plot shows the median change (red line), inner quartiles (blue box), most extreme values (black whiskers), and outliers (red +). A two-tailed paired *t* test was used to determine if the performance changes for the cohort were statistically different from zero. The *P* value of this test is given in each plot, and was less than our significance threshold 0.05 for all performance metrics. Results for each participant are shown in Additional file 1: Table S1 and Fig S1

A set of tests were performed at baseline and post-intervention to determine the effects of the exoskeleton intervention on gait. The 10MWT was used to determine changes in gait speed. Participants exhibited significantly higher walking speed *(P* value = 0.001) when compared to baseline under both self-selected (Fig. 3, top left) and fastest safe walking speed conditions (Fig. 3, top center). For self-selected gait speed, group mean improvement was 0.18 m/s and for the fastest safe gait speed there was a group mean improvement of 0.21 m/s. The minimally clinically important difference (MCID) for a 10MWT is 0.13 m/s in the older adult population. Thus, the exoskeleton intervention resulted in both a statistical and clinically significant change in gait speed. The 6MWT measures submaximal aerobic capacity and endurance. Participants covered a significantly longer distance (*P* value < 0.001) during the 6MWT post-intervention 433.7 (SD = 113.1) m compared to baseline 371.2 (SD = 94.5) m (Fig. 3, top right). The average improvement in 6MWT test after gait training with the GEMS-H was 62.5 m and the MCID for the 6MWT is 50 m [26], which means intervention resulted in a statistically and clinically significant change in aerobic capacity and endurance. Overall, these improvements in outcome measures support our hypothesis that this robotic exoskeleton-based intervention would lead to significant walking functional benefits.

### Functional balance

The BBS is a widely used clinical test that assesses an individual’s static and dynamic balance abilities. The mean BBS scores at baseline and post testing were 48 ($\mathrm{SD}=$ 7) and 52 (SD = 6) out of 56 respectively (Fig. 3, bottom left). The MCID for adults who score greater than 45/56 points on the BBS at baseline evaluation is 3.3 points [27]. The improvements observed in this study were higher than MCID and statistically significant (*P* value < 0.001). During the progression of the study, we noted a ceiling effect with the BBS in the first four participants, the FGA was added for more comprehensive evaluation of balance. The FGA was assessed for eight of the twelve participants. The mean FGA score at baseline was 17 (SD = 2.9) and increased to 23 (SD = 3.6) at post testing out of a possible 30 points, with an average change of 5.1 points (*P* value < 0.001; Fig. 3, bottom center). The MCID for the FGA is four points [28]. For the 5-times sit to stand (5xSTS), post-testing scores reduced from 15.2 (SD = 4.1) seconds at baseline to 12.3 (SD = 4) seconds following intervention *(P* value < 0.001; no MCID available; Fig. 3, bottom right).

### Number and duration of sedentary bouts

Due to sensor failure, only ten out of twelve subjects have ActiGraph data before and after the intervention, but those ten showed decreases in the number and total duration of sedentary bouts. The group’s average number of daily sedentary bouts greater than three minutes reduced from 53.7 bouts/day before intervention to 44.3 bouts/day after, which was statistically significant across subjects, with *P* value = 0.004. The total time spent in these sedentary bouts also decreased from 610.2 min/day before intervention to 497.3 min/day after, statistically significant with *P* value = 0.003. Due to sensor failure, Subject 1 only had two consecutive days of data instead of six for the pre-intervention period. Subject 1 was not excluded from the above analysis, but as a note, the *P* values shown are still < 0.05 even if Subject 1 is excluded. Please see Additional file 1: Table S3 for the daily sedentary behavior values for each available subject. Overall, these results support our hypothesis that this intervention would reduce sedentary time.

### Participant survey satisfaction levels

QUEST score was 4.5 (SD = 0.6) out of 5 for the exoskeleton use [29]. In general, users enjoyed their experience with the GEMS-H device. There were no adverse events recorded throughout the training protocol.

### Exoskeleton safety

No device-related adverse events occurred in the study. There were no device-related falls or adverse events in the study. There were no device malfunctions/breakdowns during the trial. There were no reports of any injuries to the participating older adults. Based on clinician feedback, at the end of the trial our participants had developed a collaborative trust walking and exercising with the exoskeleton.

### GEMS-H intervention compared to traditional exercise therapy

We will highlight the results of Wang 2015 as an example of traditional community based exercise therapy in the older adult population [30]. In that study, 17 subjects were selected based on their age (65 +) and ability to walk without an assistive device. In our study, 10 out of 12 participants were also able to walk without an assistive device. In Wang 2015, each participant participated in one hour sessions, three times per week, over twelve weeks. In each session, participants completed 20 min of strength training, 20 min of balance training, and 20 min of endurance training. None of these interventions included the use of an exoskeleton. Outcome measures including self-selected walking speed, fast walking speed, and 6MWT time were recorded at baseline, at an 8-week mark, and at the 12-week mark. In Table 1, we compare the baseline and 8-week outcomes of Wang 2015 with our baseline and final outcomes. At the 8-week mark of Wang 2015, their participants had received a total of 24 h of guided exercise over 24 sessions, opposed to only six hours and twelve sessions for the GEMS-H study. The GEMS-H group showed larger increases in walking speed and distance in all three categories.

**Table 1:** Mean self-selected walking speed, fast walking speed, and 6MWT distance travelled pre- and post-intervention for both our study (GEMS-H) and Wang 2015 (Traditional)

	Self-selected walking speed (m/s)		Fast walking speed (m/s)		6-Minute Walk Test (m)	
	GEMS-H	Traditional	GEMS-H	Traditional	GEMS-H	Traditional
Pre-intervention	1.03	1.03	1.42	1.38	371.1	437
Post-intervention	1.21	1.15	1.62	1.48	433.6	484
Difference	0.18	0.12	0.21	0.1	62.5	47
% Difference	17%	12%	15%	8%	17%	11%

## Discussion

This preclinical investigation was the first effort to explore the feasibility of implementing a hip exoskeleton-based functional exercise intervention in the otherwise healthy community-dwelling older adult population. Another unique aspect is that unlike traditional clinical interventions in robotics literature, which are predominantly performed in a controlled clinical or laboratory setting, in this investigation all outcome assessments and trainings were performed in a real-world community setting (senior living community). In addition, we also implemented a personalized intervention process which was a clinician-supervised, self-paced, and progressive multicomponent intervention using the exoskeleton. We hypothesized that compared to the baseline condition, implementing the GEMS-H-based multicomponent exercise intervention would lead to significant benefits in outcomes related to functional gait, balance, endurance, and reducing sedentary time. Overall, our hypotheses were accepted. Furthermore, the assessed outcome measures improved in magnitude sufficient enough to provide evidence for clinically relevant improvements through this intervention. Interestingly, these results were achieved in only twelve sessions.

With our intervention, the clinically meaningful improvements seen in gait speed, endurance, and balance have important implications as they are critical indicators of improving health state and physical frailty in the older adult population [31, 32]. While other interventions have yielded improvements in functional outcomes, the results did not meet the MCID benchmark in the same timeframe and hours of training as our study [30]. The cohorts’ average improvement in the distance covered during the 6 MWT was ~ 62.5 m, which is higher than the established MCID benchmark (50 m), signifying improved cardiovascular endurance and submaximal aerobic capacity. Furthermore, the 10MWT, a measure of gait speed, showed the largest improvement after training of all clinical outcomes measures. Walking speed is a reliable, sensitive, and specific measure correlated with functional ability and balance confidence [33]. Subject 7 increased in self-selected walking speed from 0.79 m/s to 0.83 m/s, exceeding the 0.8 m/s threshold commonly used to separate limited community ambulators from community ambulators [33]. Two out of the four participants with a starting self-selected gait speed under 1.0 m/s were able to progress to a speed greater than 1.0 m/s after intervention. Exceeding this 1.0 m/s threshold is a strong indicator of reducing fall risk [34]. One-third of community-dwelling older adults fall each year [35], and a history of falls increases the risk for recurrent falls and is a common reason for admission to a long-term care facility [36]. Reduced fall risk is associated with increased likelihood of community independence in older adults. Thus, as part of a future preventative care program, the GEMS-H device could be further studied for its capability to reduce fall risk for older adults.

Post-intervention assessment also showed that this intervention reduced the sedentary time/day in this cohort by 112 min/day on average. A reduction of 22 min of sedentary time /day in community dwelling older adults is known to be beneficial in facilitating healthy aging and reducing the risks for other aging related comorbidities [37]. Further, research suggests that sedentary behavior is an independent predictor for functional fitness in older adults [38]. Reduction in sedentary time also has implications for cardiovascular mortality [39]. We speculate that participants' improvements in endurance, balance, and strength from the GEMS-H intervention could have contributed to the reduction in sedentary bouts/day by encouraging overall physical mobility in everyday life. This is also one of the first studies in older adults to show a behavioral-based improvement post intervention for a robotic-based intervention.

The results of the current pre-clinical investigation are clinically significant because typically 50 h of traditional in-clinic exercise intervention have been prescribed in community dwelling older adults to notice clinically significant improvement in performance outcomes [20, 37, 40]. However, with a GEMS-H robotic-based multicomponent personalized intervention, we observed clinically significant improvements from just six hours of in-community training (12 session of 30 min each over 4–6 weeks). In a comparative study, 36 h of training was required to achieve improvements that did not meet established MCID. The GEMS-H intervention resulted in better results in a very similar group of community-dwelling older adults, suggesting the potential benefits of exoskeleton-based gait training opposed to traditional exercise programs. It is vital to consider the positive impact a short-duration gait training program can have on improving functional benefits and its substantial implication for healthcare costs. A short duration, community-based, supervised robotic exercise intervention may have potential implications for continued maintenance of health and a path towards successful aging. These promising initial results, which supported our hypothesis that a novel robotic exoskeleton intervention would lead to walking function benefits and decreased sedentary time, encourage a larger scale clinical trial to investigate transfer and retention of learned skills.

### Limitations

Given that this was a pilot study; the sample size is relatively small. Participants were also recruited from the same community, which also may not be representative of the whole population. Retention and follow-up were not within the scope of this study, but are recommend for future studies. Specifically, the significant reduction in sedentary time after intervention (112 min on average) is notable, but there is no data on long-term sedentary behavior changes. Including a pre-frail or frail subgroup may also be helpful in teasing out the group-specific benefits of using an exoskeleton-based intervention. A matched control group of otherwise healthy older adults that do not participate in exercise intervention, or only participate in traditional gait interventions would also strengthen the impact of our findings. The long-term effects of training and device usage on functional outcomes were not investigated in this study. The frequency and duration of the training intervention (intensity) could be specific to this cohort studied. More research with a larger cohort is recommended to validate the intensity findings. Given the positive results from this preclinical trial, a large scale trial to investigate these aspects is warranted.

### Application

Following a 12-session progressive assistance/resistance training intervention with the GEMS-H, improvements were seen across overall gait function, balance, endurance and reducing the number and duration of sedentary bouts per day in a cohort of adults 65 and older. There were no adverse events recorded throughout the training protocol. In general, users enjoyed their experience with the device. Our results show that a short-duration gait training program, using a hip powered exoskeleton (GEMS-H) delivered in-community is a safe, effective, and feasible exercise program to implement with this older adult population for their continued health state and physical well-being.

## Conclusion

In summary, our observations from this pre-clinical trial showed that in-community delivered exoskeleton-based exercise intervention in the older adult population is feasible and functionally beneficial. These findings have important implications for public health, health accessibility and physical well-being in aging populations, given that one out of every five people in the United States will be older than 65 years by the year 2030 [41]. Exercise interventions that can be efficiently delivered in a community setting are increasingly important given the evolving need to provide healthcare, even during a pandemic, where there are additional risks barriers involved for the aging population traveling to a clinical facility.

While there are presently many emerging robotic technologies (soft, rigid, hybrid, etc.), most have been trialed in controlled lab settings and designed to suit specific neurologically impaired populations. Ours is the first investigation to implement a modular hip robotic-exoskeleton, with a controller design that was focused towards older adults’ specific functional needs, and to deliver and validate the efficacy of the intervention entirely in a community setting. Our hope is that the promising results from this investigation will set the stage and serve as a guide for future large scale clinical trials in older adult population that aim to deliver a purely in-community exoskeleton-based exercise intervention for healthy aging.

## Supplementary Information

**Additional file 1: Table S1: **Age, sex, and outcome metrics for all twelve older adults in this study, reported pre- and post-intervention (PRE/POST). Subjects 2 and 10 regularly used an assistive device while walking at the time of PRE testing. *Subject 2 used a cane during the PRE testing, but did not use a cane during POST testing because they had progressed and were no longer regularly using a cane. Subject 2’s walk training was also performed without a cane, in order to challenge the participant to train their balance. **Subject 10 used a rolling walker during both PRE and POST testing. Subject 10 was trained in the GEMS-H exoskeleton both with and without the rolling walker, during tasks where it was deemed safe to do so by our physical therapists. **Table S2: **Details about the first session (FIRST), last session (LAST), and difference (Δ**)** for each subject. In general, each subject was encouraged to spend more time in resistance mode over the course of the twelve ssessions, and was also encouraged to walk with more resistance gain and less assistance gain if tolerable. Resistance gains are negative, and larger absolute values of resistance gain generate more resistance torque. As a note, six subjects were not using assistance mode at all by the last session (indicated by an Assist Time LAST column equal to zero). In this table, these subjects were considered to have an assistance gain of zero for the LAST session. **Table S3**: Sedentary bout number and duration before (PRE) and after (POST) intervention, as well as the difference per subject (Δ**). **Subjects 4 and 8 were excluded from analysis because of sensor errors that caused a lack of data. *Due to sensor failure, Subject 1 only has two days of consecutive data in the POST condition, unlike all other subjects with six. **Fig.S1**: Performance-based outcome measures from both the pre- and post-intervention tests (PRE and POST, respectively). Each boxplot shows the median (red line), inner quartiles (blue box), most extreme values (black whiskers), and outliers (red +). Each participant’s change in performance is shown with a line from their PRE to POST state (see Subject Number legend).

## Data Availability

De-identified data are available from the corresponding author upon reasonable request.

## Abbreviations

DOFC: Delayed output feedback controller
GEMS-H: Gait enhancing and motivating system-hip
10MWT: Ten meter walk test
5xSTS: Five times sit-to-stand
6MWT: Six minute walk test
BBS: Berg Balance Scale
FGA: Functional gait assessment
QUEST: Quebec user evaluation of satisfaction with assistive technology
SD: Standard deviation
MCID: Minimally clinically important difference
