# Supplementary material for: Modular hip exoskeleton improves walking function and reduces sedentary time in community-dwelling older adults
Source: J Neuroeng Rehabil. 2022 Dec 30;19:144. doi: 10.1186/s12984-022-01121-4 (PMC9801566; doi:10.1186/s12984-022-01121-4)
Supplement: Supplementary file 1 — Additional file 1: Table S1: Age, sex, and outcome metrics for all twelve older adults in this study, reported pre- and post-intervention (PRE/POST). Subjects 2 and 10 regularly used an assistive device while walking at the time of PRE testing. *Subject 2 used a cane during the PRE testing, but did not use a cane during POST testing because they had progressed and were no longer regularly using a cane. Subject 2’s walk training was also performed without a cane, in order to challenge the participant to train their balance. **Subject 10 used a rolling walker during both PRE and POST testing. Subject 10 was trained in the GEMS-H exoskeleton both with and without the rolling walker, during tasks where it was deemed safe to do so by our physical therapists. Table S2: Details about the first session (FIRST), last session (LAST), and difference (Δ) for each subject. In general, each subject was encouraged to spend more time in resistance mode over the course of the twelve ssessions, and was also encouraged to walk with more resistance gain and less assistance gain if tolerable. Resistance gains are negative, and larger absolute values of resistance gain generate more resistance torque. As a note, six subjects were not using assistance mode at all by the last session (indicated by an Assist Time LAST column equal to zero). In this table, these subjects were considered to have an assistance gain of zero for the LAST session. Table S3: Sedentary bout number and duration before (PRE) and after (POST) intervention, as well as the difference per subject (Δ). Subjects 4 and 8 were excluded from analysis because of sensor errors that caused a lack of data. *Due to sensor failure, Subject 1 only has two days of consecutive data in the POST condition, unlike all other subjects with six. Fig.S1: Performance-based outcome measures from both the pre- and post-intervention tests (PRE and POST, respectively). Each boxplot shows the median (red line), inner quartiles (blue box), most ext [file 12984_2022_1121_MOESM1_ESM.docx]

**Additional Material**

**Additional** Tables:

|  |  |  |  |  |  |  |  |  |
| --- | --- | --- | --- | --- | --- | --- | --- | --- |
| Subject Number | Age | Sex | BBS | FGA | 5xSTS | 10 MWT | 10 MWT | 6 MWT |
|  | (years) |  |  |  | (seconds) | SSV (m/s) | FV (m/s) | (m) |
| 1 | 69 | Female | 56/56 | NA | 7.73/5.73 | 1.5/1.68 | 2.05/2.52 | 520/592 |
| 2* | 74 | Male | 43/50 | NA | 19.33/17.46 | 0.915/1.09 | 1.25/1.35 | 397/443 |
| 3 | 84 | Female | 52/53 | NA | 18.63/14.31 | 1.01/1.13 | 1.47/1.66 | 441/460 |
| 4 | 66 | Female | 53/56 | NA | 11.47/6.76 | 1.22/1.63 | 1.52/1.98 | 459/582 |
| 5 | 72 | Female | 51/55 | 19/25 | 12.53/11.23 | 1.22/1.37 | 1.44/1.62 | 383/479 |
| 6 | 79 | Female | 52/55 | 17/24 | 17.12/9.86 | 1.02/1.27 | 1.56/1.75 | 316/410 |
| 7 | 74 | Female | 45/48 | 13/17 | 16.21/14.14 | 0.79/0.83 | 1.32/1.32 | 278/334 |
| 8 | 70 | Male | 46/51 | 15/20 | 18.1/15.23 | 0.71/1.1 | 0.92/1.28 | 308/321 |
| 9 | 83 | Female | 53/55 | 21/26 | 13.43/11.30 | 1.01/1.11 | 1.24/1.42 | 382/454 |
| 10** | 76 | Male | 30/34 | 16/18 | 22.44/20.35 | 0.48/0.61 | 0.65/0.75 | 169/208 |
| 11 | 72 | Female | 53/55 | 22/28 | 10.25/10.31 | 1.32/1.63 | 1.89/2.21 | 445/547 |
| 12 | 85 | Female | 45/52 | 16/22 | 16.75/11.17 | 1.24/1.15 | 1.7/1.63 | 354/374 |
|  |  |  |  |  |  |  |  |  |
|  |  |  |  |  |  |  |  |  |

**Table S1:** Age, sex, and outcome metrics for all twelve older adults in this study, reported pre- and post-intervention (PRE/POST). Subjects 2 and 10 regularly used an assistive device while walking at the time of PRE testing. *Subject 2 used a cane during the PRE testing, but did not use a cane during POST testing because they had progressed and were no longer regularly using a cane. Subject 2’s walk training was also performed without a cane, in order to challenge the participant to train their balance. **Subject 10 used a rolling walker during both PRE and POST testing. Subject 10 was trained in the GEMS-H exoskeleton both with and without the rolling walker, during tasks where it was deemed safe to do so by our physical therapists.

| **Subject Number** | **Assist Time (min)** | | | **Resist Time (min)** | | | **Assist Gain** | | | **Resist gain** | | |
| --- | --- | --- | --- | --- | --- | --- | --- | --- | --- | --- | --- | --- |
|  | **FIRST** | **LAST** | **Δ** | **FIRST** | **LAST** | **Δ** | **FIRST** | **LAST** | **Δ** | **FIRST** | **LAST** | **Δ** |
| 1 | 5 | 0 | -5 | 25 | 31 | +6 | 5 | 0 | -5 | -5 | -5 | 0 |
| 2 | 15 | 10 | -5 | 15 | 20 | +5 | 8.6 | 8 | -0.6 | -4 | -2 | +2 |
| 3 | 15 | 0 | -15 | 15 | 30 | +15 | 5 | 0 | -5 | -5 | -5 | 0 |
| 4 | 10 | 0 | -10 | 20 | 32 | +12 | 4 | 0 | -4 | -5 | -5 | 0 |
| 5 | 15 | 0 | -15 | 15 | 30 | +15 | 6 | 0 | -6 | -3 | -5 | -2.0 |
| 6 | 15 | 10 | -5 | 15 | 20 | +5 | 8 | 7 | -1 | -2 | -3 | -1.0 |
| 7 | 15 | 10 | -5 | 15 | 20 | +5 | 8.4 | 6 | -2.4 | -2.6 | -4 | -1.4 |
| 8 | 15 | 10 | -5 | 15 | 20 | +5 | 8 | 7 | -1 | -2 | -3 | -1.0 |
| 9 | 15 | 0 | -15 | 15 | 30 | +15 | 7 | 0 | -7 | -5 | -5 | 0 |
| 10 | 20 | 15 | -5 | 10 | 15 | +5 | 7 | 6 | -1 | -2 | -5 | -3.0 |
| 11 | 15 | 0 | -15 | 15 | 30 | +15 | 8 | 0 | -8 | -3 | -5 | -2.0 |
| 12 | 15 | 10 | -5 | 15 | 20 | +5 | 7 | 7 | 0 | -2 | -4 | -2.0 |
| **AVG** | **14.2** | **5.4** | **-8.8** | **15.8** | **24.8** | **+9.0** | **6.8** | **3.4** | **-3.4** | **-3.4** | **-4.3** | **-0.9** |

**Table S2:** Details about the first session (FIRST), last session (LAST), and difference (Δ**)** for each subject. In general, each subject was encouraged to spend more time in resistance mode over the course of the twelves sessions, and was also encouraged to walk with more resistance gain and less assistance gain if tolerable. Resistance gains are negative, and larger absolute values of resistance gain generate more resistance torque. As a note, six subjects were not using assistance mode at all by the last session (indicated by an Assist Time LAST column equal to zero). In this table, these subjects were considered to have an assistance gain of zero for the LAST session.

| **Subject Number** | **Mean Sedentary Time (min/day)** | | | | **Mean Sedentary Bouts (count/day)** | | |
| --- | --- | --- | --- | --- | --- | --- | --- |
|  | **PRE** | | **POST** | **Δ** | **PRE** | **POST** | **Δ** |
| 1* | 801.9 | 629.2 | | -172.7 | 69.5 | 46.0 | -23.5 |
| 2 | 790.1 | 518.6 | | -271.5 | 59.5 | 46.2 | -13.3 |
| 3 | 912.3 | 683.4 | | -228.9 | 78.8 | 56.3 | -22.5 |
| 5 | 385.2 | 309.6 | | -75.5 | 35.7 | 34.3 | -1.3 |
| 6 | 658.7 | 497.9 | | -160.7 | 47.7 | 48.2 | +0.5 |
| a7 | 726.3 | 658.2 | | -68.1 | 61.0 | 51.0 | -10.0 |
| 9 | 368.9 | 328.8 | | -40.2 | 37.2 | 33.5 | -3.7 |
| 10 | 482.3 | 320.1 | | -162.2 | 47.4 | 34.0 | -13.4 |
| 11 | 563.5 | 592.1 | | +28.6 | 68.3 | 66.7 | -1.7 |
| 12 | 412.7 | 435.3 | | +22.6 | 31.5 | 27.0 | -4.5 |
| **AVG** | **610.2** | **497.3** | | **-112.9** | **53.7** | **44.3** | **-9.3** |

**Table S3**: Sedentary bout number and duration before (PRE) and after (POST) intervention, as well as the difference per subject (Δ**).** Subjects 4 and 8 were excluded from analysis because of sensor errors that caused a lack of data. *Due to sensor failure, Subject 1 only has two days of consecutive data in the POST condition, unlike all other subjects with six.

**Additional** Figures:


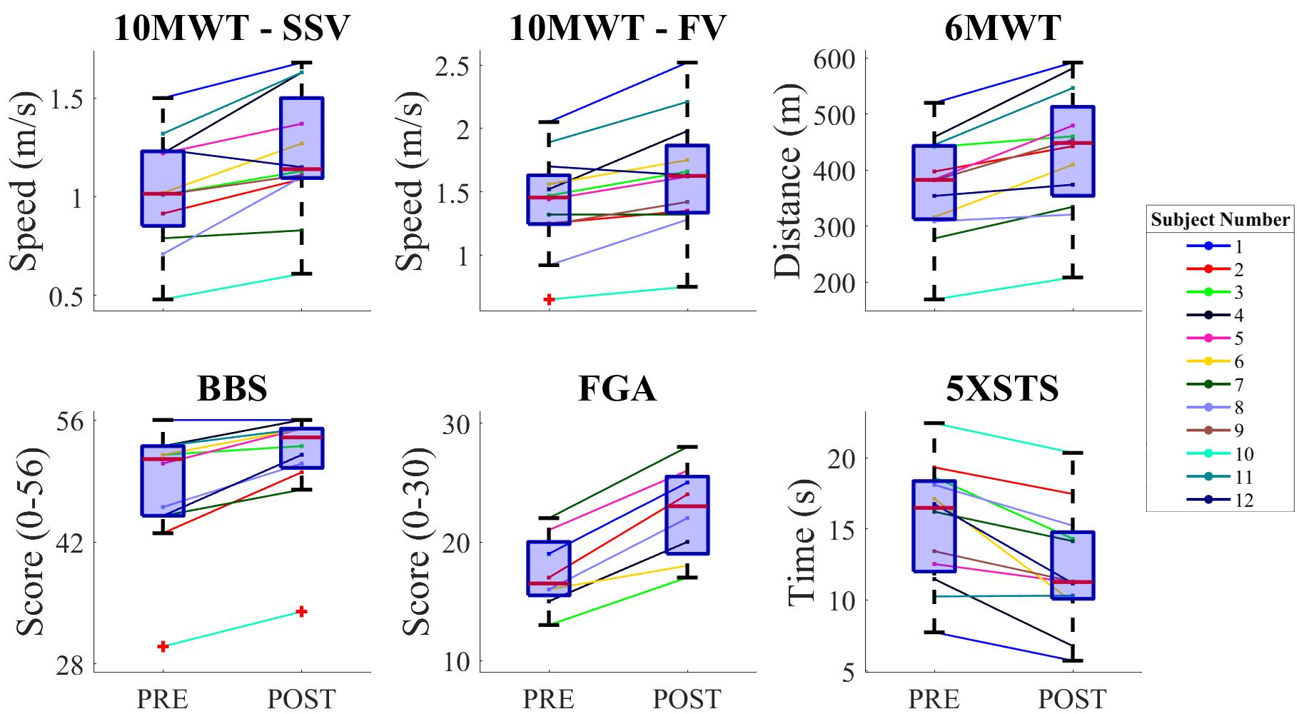


**Fig. S1**: Performance-based outcome measures from both the pre- and post-intervention tests (PRE and POST, respectively). Each boxplot shows the median (red line), inner quartiles (blue box), most extreme values (black whiskers), and outliers (red +). Each participant’s change in performance is shown with a line from their PRE to POST state (see Subject Number legend).
